# Transfer RNA Modifications in the Immune System Participate in Disease Pathogenesis

**DOI:** 10.3390/ijms27156937

**Published:** 2026-08-02

**Authors:** Xinke Ma, Hejia Gu, Yi Zhang, Hanru Jia, Peiying Li, Yingjia Wang, Jiahui Wu, Aiwei Wu, Jing Wu

**Affiliations:** Zhejiang Key Laboratory of Medical Epigenetics, Department of Immunology and Pathogenic Biology, School of Basic Medical Sciences, Hangzhou Normal University, Hangzhou 311121, China; 18757901760@163.com (X.M.); 19548905273@163.com (H.G.); 13486617505@163.com (Y.Z.); 18958510089@163.com (H.J.); unp9233@163.com (P.L.); wyj@hznu.edu.cn (Y.W.); wbhtwbzl9@163.com (J.W.)

**Keywords:** tRNA modification, immune defense, immune surveillance, immune homeostasis, immune-related diseases, intervention

## Abstract

The human immune system executes immune defense against foreign antigens, immune surveillance against mutant cells, and immune homeostasis to maintain bodily physiological equilibrium. Transfer RNA (tRNA) is an important part of gene expression regulation. Recent studies have found that aberrant tRNA modifications can disturb the function of the immune system by altering translation efficiency, including regulating immune recognition and signal transduction, as well as influencing apoptosis and autophagy. tRNA modifications are also closely related to the development and occurrence of immune-related disorders such as infections, tumors, and autoimmune diseases. By integrating current research on immune-related tRNA modifications, this review systematically summarizes the mechanisms of tRNA modifications involved in three major immune functions and their relationship with related diseases, encompassing the direct regulation of immune cells and the indirect immune effects mediated through tissue or tumor cells. In addition, some tRNA modifications can also modulate immune responses by affecting tRNA cleavage and the consequent generation of tRNA-derived small RNAs (tsRNAs). Accordingly, this review outlines promising intervention targets and unresolved research bottlenecks, facilitating follow-up investigations into tRNA modification-dependent immune regulation and translational therapeutic research.

## 1. Introduction

Transfer RNA (tRNA) is a class of non-coding RNA molecules [1] that play a crucial role in protein synthesis [2], particularly during translation. Typically composed of 70 to 90 nucleotides, tRNAs adopt a cloverleaf-like secondary structure that folds into an L-shaped tertiary structure. Their primary function is to transport specific amino acids to the ribosome and pair with the codons on mRNA, thereby translating genetic information into proteins. Recent studies have revealed that tRNAs modulate multiple physiological or pathological processes, including immune system responses [3], cellular stress reactions [4], and apoptosis [5]. Such regulatory functions of tRNAs are mediated via three major mechanisms: tRNA modifications [6], the biogenesis of tRNA-derived fragments (tRFs) [7], and differential expression of tRNAs [8]. Among these, tRNA modifications have garnered significant attention, as distinct modifications enable tRNAs to participate in diverse physiological functional alterations.

The immune system plays an important role in protecting the human body from the pathogenesis and progression of many diseases through immune defense, immune surveillance, and immune homeostasis. Among diverse tRNA-governed immune regulating mechanisms, tRNA modifications have emerged as a prominent research frontier owing to the wide variety of their chemical modification groups [9]. However, from the perspective of the immune system as a whole, there is still a lack of relevant reviews to analyze the role of tRNA modifications. Therefore, focusing on the three major functions of the immune system, defense, surveillance, and homeostasis, this review comprehensively summarizes the roles and underlying mechanisms of tRNA modification in diseases associated with immune abnormalities. This review highlights potential immune targets and prospective therapeutic strategies for the prevention and control of infectious diseases, inflammatory diseases, tumors, and autoimmune disorders.

## 2. The Role of tRNA Modifications in Immune Defense

Immune defense refers to the body’s capacity to block, recognize, and eliminate pathogens in the early stage of invasion so as to protect the body from infection and maintain homeostasis [10]. If immune defense function is compromised, the body is prone to immunodeficiency disorders, and then the risk of infection is markedly increased. Conversely, excessive activation of immune responses may trigger hypersensitivity reactions or autoimmune-mediated injury, accompanied by varying degrees of inflammatory response [11]. This section will review, from the perspectives of innate and adaptive immunity, the specific roles of tRNA modifications in immune barrier function, pathogen pattern recognition, and antigen-specific responses across different diseases.

### 2.1. QTRT1-Mediated Queuosine Modification Affects Intestinal Barrier Function

The intestinal mucosal epithelium is the main structural component that maintains intestinal barrier homeostasis and serves as an important part of immune defense. Its protective function depends on tight junctions (TJs) and epithelial cell renewal [12]. Many diseases are marked by defective barrier function, among which inflammatory bowel disease (IBD) is the most typical one.

Queuine (q), a 7-deazaguanine nucleobase, cannot be synthesized de novo by eukaryotic cells and needs to be obtained through exogenous pathways including diet and gut microbiota [13]. After entering the cell, the guanine at the wobble position 34 (G34) within the anticodon of specific tRNAs is replaced with queuine (q), yielding Queuosine-modified tRNA (Q-tRNA), which is catalyzed by the eukaryotic tRNA-guanine transglycosylase complex, consisting of the catalytic subunit queuine tRNA-ribosyltransferase catalytic subunit 1 (QTRT1) and the auxiliary subunit queuine tRNA-ribosyltransferase accessory subunit 2 (QTRT2) [14,15]. Q-tRNA modification can affect the decoding of related codons and protein translation, and may be involved in cell proliferation, stress adaptation, and the maintenance of epithelial barrier homeostasis [13,16].

Claudin family proteins are important components of TJs, among which claudin-2 can form paracellular channels and thereby increase epithelial permeability [17], whereas claudin-5 helps to maintain TJ tightness and barrier stability. Therefore, increased claudin-2 expression and decreased claudin-5 expression can change the composition of TJ proteins and impair intestinal epithelial barrier function [18]. In addition to TJ protein composition, the maintenance of intestinal epithelial barrier also depends on continuous epithelial cell renewal. The Wnt/β-catenin signaling pathway, where β-catenin functions, is an important regulatory axis to maintain intestinal crypt cell proliferation and epithelial homeostasis [19].

Based on the barrier-maintenance mechanisms described above, previous studies have further investigated the associations of changes in QTRT1 expression and Q-tRNA modification with IBD-related epithelial renewal defects and impaired TJ function. Zhang et al. observed downregulated QTRT1 expression in intestinal mucosal samples from IBD patients, and found similar changes in DSS-induced colitis and IL-10-deficient mouse models: reduced QTRT1 expression was accompanied by increased claudin-2 expression and decreased β-catenin and claudin-5 expression [16]. Subsequently, the authors observed concordant changes in cell proliferation and TJ protein expression in QTRT1-knockdown human intestinal epithelial cells and QTRT1-knockout mice, further supporting the link between QTRT1 deficiency and suppressed epithelial renewal as well as TJ dysfunction [16]. Based on the existing evidence, QTRT1 deficiency may lead to insufficient Q-tRNA modification, and is related to the inhibition of intestinal epithelial cell proliferation and abnormal expression of TJ proteins. However, whether QTRT1 deficiency directly regulates the translation of β-catenin or claudin-related proteins has not yet been fully confirmed.

In addition, the chronological sequence and causal relationships between the downregulation of QTRT1 expression, insufficient Q-tRNA modification, and barrier damage in IBD remain to be further clarified. Given queuine supply is affected by diet and gut microbiota [13], future studies could modulate the status of microbiota or queuine availability, measure QTRT1 expression and the levels of Q modification in specific tRNAs, and assess changes in epithelial cell renewal, TJ protein expression, and mucosal inflammation. These approaches may help determine whether reduced QTRT1 expression and the impaired Q-tRNA modification are the early factors contributing to barrier damage in IBD, or secondary changes arising from persistent inflammation. Moreover, through QTRT1 functional intervention, quantitative analysis of Q-tRNA modification, and combining translatomics and proteomics, the mechanisms of QTRT1-dependent Q-tRNA modification abnormality involved in intestinal epithelial barrier injury may be further elucidated.

Notably, a recent study has also revealed that TRMT6 deficiency inhibits intestinal epithelial cell proliferation by disrupting translational regulation associated with tRNA m^1^A modification, reducing MYC protein synthesis, and exacerbating DSS-induced intestinal mucosal barrier damage [20]. These findings further extend the research scope of tRNA modification involved in intestinal epithelial renewal and barrier homeostasis and reflect the rapid development of this field.

### 2.2. Gm18 Modification of tRNA Suppresses Innate Immune Recognition and Type I Interferon Responses via TLR7

The traditional view holds that innate immune cells can distinguish endogenous from exogenous RNA by sensing differences in RNA structure and modification status [21]. Previous studies have shown that RNA can induce immune responses through Toll-like receptors such as TLR3, TLR7, and TLR8, while certain natural nucleoside modifications can weaken receptor-mediated RNA recognition and subsequent downstream signaling. For instance, the 2′-O-methylation (2′-O-Me) can attenuate TLR7-mediated immune responses [22].

Gm18 denotes 2′-O-methylation of the ribose moiety of guanosine at position 18 in bacterial tRNA, which is usually catalyzed by Gm18-2′-O-methyltransferase (TrmH). Jöckel et al. found that the Gm18 modification of tRNATyr in *E. coli* displayed TLR7-antagonistic activity, interfering with tRNA binding to TLR7, thereby suppressing IFN-α production by plasmacytoid dendritic cells (pDCs) [23]. In addition, the Gm18-containing tRNA^Tyr^ sequence inhibited bacterial RNA-induced activation of human CD14^+^ monocytes, reduced IκBα phosphorylation and degradation, and decreased p38 MAPK and ERK1/2 phosphorylation, thereby lowering the production of proinflammatory cytokines such as TNF, IL-6 and IL-12p40 [24]. Further experiments using trmH-deficient *E. coli* showed that tRNA lacking Gm18 modification had an enhanced capacity to activate TLR7 and induce IFN-α. By contrast, in vitro Gm18 methylation attenuated the immune-stimulatory activity of *E. coli* tRNA [23].

Meanwhile, the influence of Gm18 on TLR7 recognition is also shaped by the local structure of the tRNA D-loop and the composition of adjacent nucleosides. Using synthetic tRNA variants, Kaiser et al. found that TLR7-mediated IFN-α production was further reduced when Gm18 and a downstream purine formed a specific dinucleotide arrangement [25]. This result indicates that the modulation of tRNA immunogenicity by Gm18 depends on the surrounding sequence and structural context. High-throughput sequencing by Galvanin et al. further revealed that 2′-O-Me modification of bacterial tRNA varied dynamically under stress conditions such as nutrient limitation and antibiotic exposure [26], suggesting that bacteria may impact the host’s recognition of bacterial RNA and subsequent inflammatory responses by adjusting tRNA modification patterns. However, whether Gm18 modification ultimately weakens host defense may also depend on bacterial species, tRNA types, and host cell types.

Based on the aforementioned studies, the local 2′-O-Me modification of tRNA represented by Gm18 may be a key modification feature influencing host TLR7 recognition of bacterial tRNAs. Studies by Robbins et al. demonstrated that RNA modified with 2′-O-Methyl can inhibit the induction of IFN-α and IL-6 by other RNAs capable of activating TLR7, and exhibited TLR7 antagonistic effects in both in vivo and in vitro models [27]. Although this study did not focus on tRNA, the results suggest that 2′-O-Me modification not only reduces the intrinsic immune-stimulatory capacity of RNA but may also suppress responses triggered by other TLR7 agonists, thereby providing complementary evidence for the TLR7-antagonistic effect of Gm18-modified tRNA.

Because TLR7 is localized to endosomal membranes, bacterial tRNA carrying Gm18 modification must first be internalized by pDCs and trafficked to receptor-containing endosomes before it can affect TLR7-mediated IFN-α response [28]. Future studies could therefore examine the roles of Gm18 modification in bacterial tRNA uptake and endosomal delivery, together with its regulation of pDCs IFN-α responses, to clarify its biological significance in innate immune defense and inflammatory regulation.

### 2.3. tRNA I34 Modification Enhances B Cell-Mediated Adaptive Immune Responses by Improving Antibody Translation Efficiency

Beyond its involvement in host innate immunity, tRNA modification can also influence antibody synthesis during adaptive immune responses. As key effector molecules of humoral immunity, their continuous production and sustained peak concentration depend on the protein synthesis capacity of plasma cells [29].

Studies have shown that antibody-coding sequences preferentially use certain codons during translation, but the supply of fully cognate tRNAs is insufficient [30]. This mismatch between codon usage and tRNA availability may therefore affect the efficiency of antibody synthesis. Adenosine at position 34 of the tRNA anticodon loop can be converted to inosine (I34) by the ADAT2/ADAT3 complex, thereby broadening the wobble-decoding capacity of a single tRNA for multiple synonymous codons [31,32] and alleviating the mismatch between antibody codon preference and tRNA availability.

In *Adat2*-knockout mice, peripheral blood B cells were significantly reduced, suggesting that ADAT2-mediated I34 modification may impede B cell development and impair their survival in the periphery [29]. Giguère et al. generated an Igh^I34^/WT mouse model enriched in I34-dependent codons by replacing synonymous codons in antibody variable-region genes. They found that increased use of these codons raised antibody expression and strengthened the competitive advantage of B cells during bone marrow development and in peripheral immune organs [30]. Moreover, antibody-secreting cells exhibited higher I34 levels than naïve B cells and showed a greater dependence on I34-dependent translation [30]. In light of Lyu et al.’s study on I34 modification regulating codon-dependent translation kinetics and affecting protein expression profiles [33], these observations indicate that I34 modification may help maintain antibody production after B-cell differentiation into plasma cells by improving translational efficiency.

Importantly, improved antibody translation efficiency does not necessarily indicate a stronger adaptive immune response. Effective humoral immunity also depends on antibody affinity, antigen specificity, germinal-center reactions, and memory B-cell formation [34,35]. Accordingly, I34 modification mainly provides translational support for the large-scale synthesis of antibodies, but whether it really enhances effective humoral immunity needs to be further verified from the quality of antibodies, the outcome of B cell differentiation and the disease background. Further studies are needed to compare the different effects of I34 modification on the generation of protective and abnormal antibodies.

Overall, tRNA modifications can influence the processes by which the body resists, recognizes, and clears foreign substances. Aberrant tRNA modifications may disrupt immune defense and contribute to infection, inflammation, and immune-related diseases [9]. Future strategies may precisely regulate these modifications by controlling the supply of modification precursors, designing and delivering tRNAs with specific modifications, and modulating the activity of tRNA-modifying enzymes to recover the impaired immune defense or suppress the abnormal responses. Compared with conventional approaches based on broad immunosuppression, such interventions may more selectively regulate aberrant immune processes while limiting their effects on normal host defense. Nevertheless, further validation across different cell types and disease stages is needed to ensure that normal resistance to infection is not compromised.

## 3. The Role of tRNA Modification in Immune Surveillance

The body’s defense process, known as immune surveillance, constantly identifies and gets rid of aberrant elements, like tumor cells that result from gene mutations and senescent or dead cells [3]. The immune surveillance function of the body can promptly eliminate newly emerging malignant cells, thereby preventing tumor development. However, when immune surveillance is impaired, tumor cells can evade it through various mechanisms, leading to tumor growth and metastasis.

### 3.1. METTL1 and WDR4-Mediated tRNA m^7^G Modification Promotes Tumorigenesis

N6-methyladenosine methyltransferase-like 1 (METTL1) and WD repeat domain 4 (WDR4) form a complex that jointly catalyzes site-specific methylation on tRNAs [36]. Within this methyltransferase complex, METTL1 serves as the m^7^G catalytic enzyme, while WDR4 provides structural stabilization [37,38]. The METTL1/WDR4 complex catalyzes N7-methylation at the G46 position of tRNA, producing N7-methylguanosine (m^7^G) [39]. This modification preferentially enhances translation of mRNAs bearing specific codons and protects tRNAs from cleavage, thereby reducing small RNA fragment generation. Consequently, it affects translation of proteins including transforming growth factor-β2 (TGF-β2), pro-inflammatory factors, anti-inflammatory factors, and the regulatory-associated protein of mammalian target of rapamycin (RPTOR). Alterations in these immune system-specific proteins modulate a range of immune-related biological activities, causing dysfunction in CD4^+^ and CD8^+^ T cells, macrophages, and other immune surveillance components. This creates an immunosuppressive microenvironment that ultimately enables tumor immune evasion.

#### 3.1.1. METTL1 Enhances TGF-β2 Protein Synthesis to Promote HCC Recurrence

Radiofrequency ablation (RFA) represents a crucial local treatment for hepatocellular carcinoma (HCC), inducing tumor cell death through thermal energy while triggering complex immune responses. However, the recurrence rate remains as high as with all other treatment modalities for HCC. TGF-β2, an important member of the TGF-β superfamily, exerts anti-inflammatory and immunosuppressive effects. Studies have demonstrated that METTL1 expression is upregulated in recurrent HCC following RFA, accompanied by increased CD11b^+^CD15^+^ polymorphonuclear myeloid-derived suppressor cells (PMN-MDSCs) and reduced CD8^+^ T cells. The underlying mechanism involves heat-induced upregulation of METTL1, which promotes m^7^G modification and enhances TGF-β2 translation. This induces accumulation of immune-suppressive cells such as CD11b^+^ CD15^+^ PMN-MDSCs and decreases CD8^+^ T cell infiltration, establishing a local immunosuppressive microenvironment [40]. Such an environment facilitates HCC cell growth and metastasis, contributing to post-RFA recurrence.

#### 3.1.2. METTL1 Activates Inflammatory Factor Synthesis to Promote ICC Progression

Programmed cell death protein-1 (PD-1) serves as a critical negative immune checkpoint molecule that maintains immune tolerance, prevents autoimmune reactions, and regulates T cell function [41,42]. Tumor cells extensively express PD-1 to inhibit the T cell PI3K/AKT/mTOR signaling pathway, Tumor cells extensively express PD-1 to inhibit the T-cell PI3K/AKT/mTOR signaling pathway, leading to T-cell functional exhaustion and proliferation arrest, thereby achieving immune evasion [43]. In vivo experiments have shown that METTL1-mediated m^7^G tRNA modification promotes translation of human CXCL8 and mouse CXCL5, inducing accumulation of PMN-MDSCs in the tumor immune microenvironment (TIME). This restricts CD4^+^ and CD8^+^ T cell proliferation and anti-tumor activity, diminishes the efficacy of PD-1 blockade, and consequently promotes intrahepatic cholangiocarcinoma (ICC) progression [44].

#### 3.1.3. METTL1-Mediated tRNA m^7^G Modification Protects tRNAs from Cleavage, Reducing Immune Effects Triggered by Small tRNA Fragments

In prostate cancer (PCa), METTL1-mediated tRNA m^7^G modification protects tRNAs from cleavage into inhibitory small ncRNAs, particularly 5′-terminal oligoguanine-containing tRNA fragments (5′TOGs). IRF9 (interferon regulatory factor 9) and ISG15 (interferon-stimulated gene 15) constitute core components of the type I interferon signaling pathway, functioning as transcriptional regulators and protein modification molecules, respectively. STAT1, a member of the signal transducers and activators of transcription (STAT) family, is upregulated when 5′TOGs displace translation regulators from mRNA caps, thereby enhancing IRF9 and ISG15 translation and activating the IFN pathway. Conversely, tRNA m^7^G modification suppresses this process by reducing inflammatory cytokine release and inhibiting specific immune cell activation, thereby suppressing anti-tumor effect of the immune system [45].

Tumor-associated macrophages (TAMs) represent key components of the tumor microenvironment (TME), typically exhibiting M2-type macrophage gene expression characteristics [46] and broadly promoting tumorigenesis and progression [47]. TAMs not only facilitate cancer cell proliferation, invasion, angiogenesis, and immune tolerance but also significantly reduce tumor cell sensitivity to conventional therapies [48,49]. Inhibiting METTL1 in tumor cells can polarize TME immune cells toward cytotoxic anti-tumor phenotypes and initiate immune cell anti-tumor effects. Specifically, METTL1-mediated tRNA modification likely suppresses the IFN pathway, inducing down-regulation of anti-inflammatory cytokines including macrophage colony-stimulating factor (M-CSF), IL-10, and IL-13. This drives macrophage polarization toward M2-like macrophages and restricts their anti-tumor functions [45].

#### 3.1.4. METTL1/WDR4 Complex Indirectly Suppresses Immune Surveillance by Regulating Tumor Cell Autophagy to Promote Tumor Development

Autophagy is an evolutionarily conserved intracellular degradation process wherein double-membrane autophagosomes engulf cytoplasmic components for lysosomal degradation and recycling, maintaining cellular homeostasis. Impaired autophagy reduces antigen presentation and immunostimulatory cytokine release, failing to effectively activate tumor-specific CD8^+^ T cells and initiate systemic anti-tumor immune responses [50].

**Figure 1:**
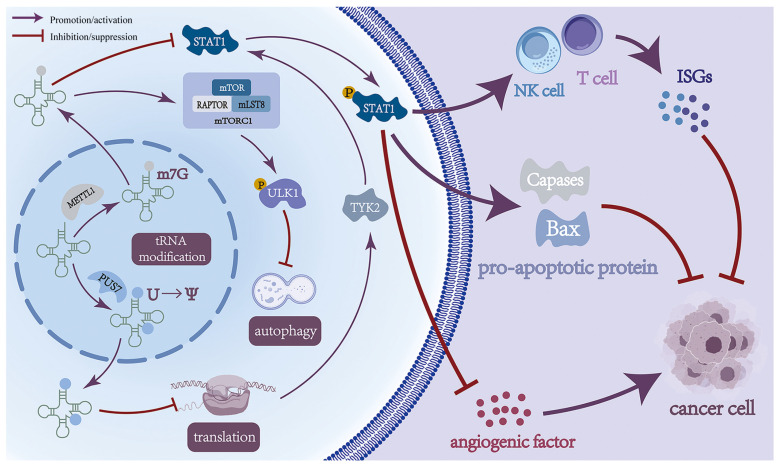
Pathways related to tRNA modification involved in tumor immune evasion. tRNA undergoes pseudouridylation mediated by PUS7, inhibiting the translation of specific codons and leading to reduced Tyrosine kinase 2 (TYK2) expression. This can be linked to (or occur separately from) the inhibition of signal transducers and activators of transcription 1 (STAT1) expression caused by METTL1-mediated tRNA m^7^G modification, thereby suppressing the TYK2-STAT1 pathway, inhibiting the IFN pathway, and ultimately causing immune escape either directly or through reduced tumor suppressor factor levels. Additionally, by increasing protein of mammalian target of rapamycin (RPTOR) translation, m^7^G alteration can activate the mTOR pathway, phosphorylate Unc-51-like autophagy-activating kinase 1 (ULK1), block autophagy, and aid immune evasion. In the figure, arrows indicate promotion or activation, whereas lines with T-shaped ends indicate inhibition or suppression.

The RPTOR protein exhibits elevated levels of m^7^G modification, making it a key protein that positively regulates the mTOR signaling pathway. In esophageal squamous cell carcinoma (ESCC) tissues, the METTL1/WDR4 complex upregulates RPTOR expression, mediating mTOR pathway-dependent phosphorylation of Unc-51-like autophagy activating kinase 1 (Ulk1) at Ser758 (pULK1), thereby negatively regulating autophagy (Figure 1) [51] and promoting ESCC progression [52].

Additionally, studies have revealed significantly elevated and positively correlated expression of METTL1 and WDR4 in head and neck squamous cell carcinoma (HNSCC). This research demonstrated that Mettl1 promotes PI3K protein translation through m^7^G modification, subsequently increasing AKT and mTOR phosphorylation levels. This cascade inhibits apoptosis, drives G1-to-S phase cell cycle transition, suppresses autophagy, promotes tumor cell survival and exacerbates disease progression [39].

Taken together, METTL1 promotes translation of mTOR signaling pathway-related proteins in an m^7^G codon-dependent manner, thereby suppressing tumor cell autophagy, inhibiting activation of the tumor immune surveillance system, and facilitating tumor cell immune evasion.

### 3.2. ALKBH3-Mediated tRNA Demethylation Impairs Immune Surveillance Efficacy by Inhibiting Tumor Cell Apoptosis

Previous studies have identified angiogenin (ANG) as an enzyme involved in tRNA cleavage [53], while tRNA methylation protects against such cleavage [54]. Human AlkB homolog 3 (ALKBH3) functions as a tRNA demethylase that removes methyl groups from N1-methyladenosine (m^1^A) and 3-methylcytidine (m^3^C) in tRNAs both in vitro and in vivo [55]. ALKBH3-mediated demethylation renders tRNAs more susceptible to ANG cleavage, leading to generation of tRNA-derived small RNAs (tsRNAs). Cytochrome c (Cyt c) serves as a core initiator of apoptosis, whose release is a critical step when immune effector cells such as CTLs and NK cells induce target cell apoptosis [56,57]. tsRNAs directly bind to cytochrome c, blocking its release and inhibiting apoptosis [55]. Under normal conditions, cells with genetic mutations, including tumor cells, are eliminated through apoptosis, which is a key aspect of immune surveillance. The ALKBH3-mediated mechanism of blocking apoptosis allows potentially malignant cells to evade immune system recognition and clearance, thereby promoting tumorigenesis and progression [55].

### 3.3. PUS7-Regulated tRNA Pseudouridylation Suppresses Anti-Tumor Immune Responses

Tyrosine kinase 2 (TYK2), a member of the Janus kinase (JAK) family, is activated by IL-12, IL-23, and type I interferons (IFNs) to regulate signal transduction and immune cell differentiation [58]. Within the IFN signaling pathway, TYK2 cooperates with STAT1 to activate NK and T cell functions, participating in positive regulation of cellular immune responses [59].

The pseudouridine modification (Ψ50) of uridine at position 50 (U50) in tRNA-Arg-CCG-2-1 is selectively catalyzed by pseudouridine synthase 7 (PUS7), which inhibits the TYK2-STAT1 pathway and suppresses TYK2 expression in glioma stem cells (GSCs). This process contributes to tumor immune evasion and promotes tumor cell survival (Figure 1) [60].

In summary, METTL1/WDR4 complex-mediated m^7^G modification, ALKBH3-mediated demethylation, and PUS7-mediated pseudouridylation influence tRNA stability and functional properties, thereby regulating translation levels of key molecules in TGF-β2, inflammatory factors, mTOR pathway-related proteins, apoptosis-related proteins, and the TYK2-STAT1 signaling pathway. tRNA chemical modifications exert critical roles in immune surveillance by regulating specific gene expression, translation efficiency, protein synthesis, and small tRNA fragment generation, thereby influencing tumorigenesis, immune evasion, and anti-tumor immune responses. Thus, tRNA modifications function as post-transcriptional regulatory hubs that play indispensable roles in gene expression regulatory networks through their effects on translation. These findings offer novel perspectives for tumor-targeted therapy: tRNA modification enzymes represent potential therapeutic targets. Small-molecule inhibitors targeting METTL1-WDR4, ALKBH3, or PUS7, such as HUHS015 and its derivatives, as well as Rhein [61,62], may block tumor-specific translation programs and restore anti-tumor immune responses. By remodeling the tumor immune microenvironment and inhibiting pro-tumorigenic tRNA modifications, dual immune activation effects could be achieved. On one hand, reducing translation of immunosuppressive factors such as TGF-β2 decreases Treg cell and M2 macrophage infiltration, thereby relieving immunosuppression. On the other hand, promoting translation efficiency of the TYK2-STAT1 pathway and type I interferon signaling to enhance DC cross-presentation and cytotoxic functions of CD8^+^ T cells and NK cells, thereby synergistically boosting anti-tumor immune responses. Finally, combined application of tRNA modification inhibitors with immune checkpoint inhibitors, metabolic interventions, or epigenetic drugs may produce synergistic effects to overcome immunotherapy resistance. Despite challenges regarding specificity and dynamic regulatory complexity, precise targeting of tRNA modifications represents a promising next-generation approach for tumor immunotherapy, potentially driving the paradigm shift in cancer treatment to “tumor-specific translation regulation” strategies.

## 4. The Role of tRNA Modification in Immune Homeostasis

Immune homeostasis refers to a physiological function of the body to maintain the homeostasis of the internal environment by timely eliminating damaged, aging, and degenerated cells, as well as foreign substances such as immune complexes. The maintenance of immune homeostasis generally requires three key conditions: appropriate activation of immune cells when needed, effective execution of clearance functions, and timely termination of inflammation to promote tissue repair. Abnormality in any step may cause autoimmune diseases. Autoimmune diseases are a class of disorders in which the body’s immune system mistakenly recognizes its own tissues or cells as foreign substances, resulting in immune responses against self-antigens, leading to inflammation, tissue damage, or dysfunction [63]. At present, the research on immune homeostasis predominantly focuses on classic autoimmune diseases, and the underlying mechanisms are mostly explored at the level of immune cells and microRNAs (miRNAs) [64]. In contrast, the role and mechanistic contributions of tRNA modifications in this process remain poorly understood. Therefore, we focus on the key conditions for maintaining immune homeostasis and explore the role and mechanism of tRNA chemical modification in autoimmune diseases and other diseases related to immune homeostasis imbalance, aiming to facilitate the prediction, diagnosis, treatment, and prognosis of these diseases.

### 4.1. The 6-TG Modification of tRNA Restricts Aberrant Activation of T Cells

**Figure 2:**
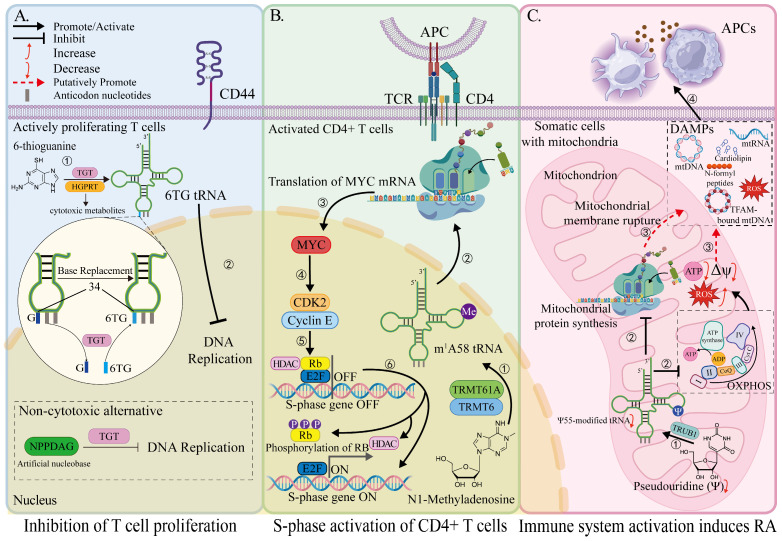
Mechanistic pathways of tRNA modifications in immune homeostasis. (**A**) In actively proliferating T cells, ➀ 6-thioguanine (6TG) modifies tRNA through TGT, ➁ and 6TG-containing tRNA inhibits cell proliferation by interfering with DNA replication processes by mechanisms that have not been fully elucidated. (**B**) In activated CD4^+^ T cells, ➀ A58 of tRNA is converted to m^1^A by TRMT61A and TRMT6 in the nucleus ➁ and promotes the transcription of extranuclear MYC protein. ➂–➃ Upregulated MYC promotes the formation of CDK2-Cyclin E complex, ➄–➅ and finally drives the cell into S phase by regulating the Rb-E2F pathway. (**C**) In somatic mitochondria, ➀ when TRUB1-mediated mitochondrial tRNA Ψ55 modification is absent, ➁ mitochondrial protein synthesis may be impaired, leading to decreased OXPHOS function and mitochondrial structural and functional impairment, ➂–➃ which may promote the release of contained damage-associated molecular patterns (DAMPs) from mitochondria and activate the immune system to induce rheumatoid arthritis (RA).

Multiple sclerosis (MS) is an autoimmune disease that affects the central nervous system. It is characterized by the rapid proliferation and differentiation of autoreactive T cells that penetrate the central nervous system [65], mainly manifested as abnormal attacks on the myelin sheaths of neurons in the brain and spinal cord by the autoimmune system [66]. 6-thioguanine (6TG) is a potent cytotoxic agent with anti-proliferative effects [67], which has been shown to serve as a substrate for tRNA guanine transglycosylase (TGT) in eukaryotic cells [68]. Its integration into tRNA enables the selective inhibition of aberrant proliferation in autoreactive T cells (Figure 2A). Mechanistic studies have demonstrated that the 6TG modification of tRNA in regenerating liver or rapidly proliferating cells suppresses the proliferation and activation of effector immune cells without affecting the quiescent and post-proliferative cell populations, thereby reducing the activity of over-proliferating autoreactive T cells and finally exerting therapeutic effects on MS [69].

However, the anti-proliferative effect of 6TG can also be achieved through nucleotide conversion catalyzed by hypoxanthine-guanine phosphoribosyltransferase (HPRT). However, HPRT also causes the cytotoxicity of 6TG, which restricted the application of 6TG due to safety concerns [70]. In contrast, the artificial base NPPDAG, which is the substrate of TGT but inert as a substrate for HPRT, can rapidly and completely relieve symptoms in the MS mouse model with no cytotoxicity [69]. Mechanistically, NPPDAG selectively inhibits the proliferation of antigen-specific effector T cells and memory T cells without affecting naive T cells, providing a safer new therapeutic strategy for MS (Figure 2A).

The 6TG modification of tRNA maintains immune homeostasis by limiting the abnormal immunity of T cells to their own tissues, and this mechanism may play a role by regulating autoreactive T follicular helper (Tfh) cells. This finding reveals the potential value of purine modification therapy in autoimmune diseases and may be extended to T cell dysfunction diseases such as systemic lupus erythematosus (SLE), but this hypothesis remains to be verified.

### 4.2. Defective m^1^A58 Modification Results in CD4^+^ T Cell Dysfunction

CD4^+^ T cells are helper T cells. Their main role is to help other immune cells recognize and fight against pathogens [71], and their inappropriate differentiation can lead to progression of inflammatory and autoimmune diseases [72]. Distinct CD4^+^ T cell subsets play protective or pathogenic roles in SLE [73], rheumatoid arthritis (RA) [74], psoriasis [75], and Crohn’s disease (CD) [76].

N1-methyladenosine (m^1^A) is a methylation modification of the first nitrogen atom of adenine in RNA molecules. It is usually located at the 58th position of tRNA, named m^1^A58. The methyltransferases TRMT61A and TRMT6 collaboratively catalyze the m^1^A58 modification of a subset of early-expressed tRNA [77,78]. Deficiency of TRMT61A reduces tRNA m^1^A58 modification, thereby down-regulating MYC protein expression at the translational level. MYC is a transcription factor whose role is to promote rapid metabolism, reprogramming, and cell proliferation of transformed cells [79,80]. Deficiency of MYC leads to decreased protein expression of cyclin-dependent kinase 2 (CDK2) and cyclin E, thereby arresting cell cycle S-phase progression through the MYC-CDK2 axis. Ultimately, it suppresses aberrant proliferation and over-activation of CD4^+^ T cells (Figure 2B) [81]. Consistent with these findings, reducing TRMT61A expression can inhibit the proliferation and over-activation of CD4^+^ T cells [82].

Based on this mechanism, TRMT61A can serve as a target for autoimmune diseases such as SLE: targeting m^1^A58 modification may correct the imbalance of immune homeostasis and restore immune balance by inhibiting the proliferation of CD4^+^ T cells.

In addition, abnormal expression of tsRNAs, which can be regulated by tRNA modification, affects Th1/Th2 differentiation, leading to Th1/Th2 imbalance, and then induces SLE [83], but the specific molecular mechanism needs to be further studied.

### 4.3. TRUB1-Mediated Mitochondrial tRNA Ψ55 Modification Regulates T Cell Metabolism

The Ψ55 modification of mitochondrial tRNA belongs to the pseuduridylation of tRNA, which is catalyzed by TRUB1 and plays an important role in maintaining the normal function of mitochondrial tRNA metabolism, mitochondrial protein translation, and the oxidative phosphorylation (OXPHOS) system [84]. Studies have shown that damage to mitochondrial structure and function can lead to an increase in mitochondrial damage-associated molecular patterns (DAMPs) in plasma [85,86]. Therefore, based on the indirect evidence provided by the studies cited above, we speculate that the loss or impairment of TRUB1-mediated mitochondrial tRNA Ψ55 modification may affect mitochondrial protein translation and OXPHOS function, induce mitochondrial structural and functional impairment, and further promote the release of mitochondrial-derived DAMPs [84,85,86,87,88] (Figure 2C). However, direct evidence demonstrating that abnormal TRUB1-mediated mitochondrial tRNA Ψ55 modification can directly induce DAMP release in RA is still lacking, and its specific role requires further validation.

### 4.4. Elp3/Ctu1-Mediated Modification at the U34 Site Regulates Macrophage Polarization and Inflammatory Resolution

The Elongator complex subunits Elp3 and Cytosolic thiouridylase (Ctu1) regulate mTORC2 activation by modifying U34 in tRNA, thereby inhibiting M1 polarization. Elp3 catalyze the modification of U34 of tRNA with 5-methoxycarbonyl methyl (mcm^5^) or 5-carbamyl methyl (ncm^5^), followed by 2-thiylation (s^2^) modification at the same position catalyzed by of Ctu1, jointly enhancing tRNA function [89]. In myeloid cells, M1 activation down-regulates Elp3 expression and inhibits mcm^5^S^2^ U34 tRNA modification, which in turn impairs the synthesis of Ric8b (the key protein to activate mTORC2) and weakens mTORC2 activation. mTORC2 regulates the polarization of macrophages to M2 type by phosphorylating Akt at Ser473 to phosphorylate substrate FoxO1 and inhibit the production of pro-inflammatory factors [90].

In M1 macrophages, Elp3 deficiency enhances the proinflammatory response, thereby exacerbating the symptoms of experimental colitis in mice. However, in M2 macrophages, Elp3 deficiency inhibited the phosphorylation of STAT6 and mTORC2 pathway when macrophages were stimulated by IL-4, IL-13 or both, and finally inhibited M2 macrophage polarization [91]. Therefore, we conclude that U34 modification of tRNA can regulate macrophage polarization (M1/M2 balance), coordinate pro-inflammatory and anti-inflammatory effects, participate in the maintenance of immune homeostasis and regulate immune local balance, and thus play a key role in the development of immune diseases such as colitis.

In summary, the main chemical modification of tRNA affecting immune homeostasis is methylation. These modifications can lead to excessive activation or functional defects of T cells, B cells and other immune cells, or abnormal induction of inflammation. Whether these tRNA modifications and their corresponding enzymes can be used as therapeutic targets for these diseases is also a topic worthy of further exploration.

## 5. Challenges in tRNA Research

Current studies on tRNA modifications have advanced our understanding of the mechanism and consequences of tRNA modification in immunity and immune-related diseases; however, many ambiguities remain in this field, which can be partly attributed to the defects in detection techniques, unbalanced experimental evidence, and ambiguous key mechanisms.

### 5.1. Limitations in Detection Technologies

The existing tRNA modification detection approaches, such as tRNA sequencing and mass spectrometry, suffer from inaccurate site localization, unreliable quantification, and missed identification of low-abundance modifications [92]. In vitro sample manipulation can easily lead to tRNA degradation and the loss of modification, making it difficult to reflect the true level in vivo [93]. Lack of tissue-or cell type-specific detection methods hinders the comprehensive dissection of the functional mechanism of tRNA modification in immune cells [93].

### 5.2. Uneven Distribution of Research Evidence

Most of the current studies focus on the association between a single modification and one specific disease, with marked discrepancies in the source and reliability of supporting evidence. For example, the QTRT-Queuosine-IBD axis has been verified by both animal and clinical samples, while the effect of TRUB1-mediated tRNAΨ55 modification on T cell metabolism and of ALKBH3/tRNA demethylation in tumor immune escape are mostly indirect evidence and speculative conclusions, lacking in vivo experiment verification and clinical evidence.

### 5.3. tRNA Modification and tsRNA Generation Association Are Ambiguous

Some studies suggest that tRNA modification affects tsRNA production (for example, ALKBH3 demethylation enhances ANG cleavage and promotes tsRNA production) [60], but the relationship between tRNA modification, cleavage site, and tsRNA functional mechanism is unclear. At the same time, whether the immunity is mediated by tRNA, tsRNA or the combination of the two is still lacking experimental verification.

## 6. Summary and Outlook

In summary, tRNA modifications frequently impair the immune system’s normal function, thereby triggering diseases (Table 1) [94]. This offers a novel perspective for understanding disease pathogenesis. While much research on disease onset and progression has focused on tumors, neurological disorders, and cardiovascular diseases. Very few studies have linked tRNA modifications to immune system dysfunction; this paper serves as an excellent supplement to this field.

In previous sections, we have elucidated the close relationship between tRNA modifications and tumor immune evasion [44]. Tumor immune evasion represents a main cause of tumorigenesis and progression. This sufficiently demonstrates that tRNA modifications inevitably undergo scrutiny by the immune system during disease initiation. Therefore, gaining a deeper understanding of the mechanisms underlying tRNA modification changes can help us comprehend more disease etiologies and identify more direct and effective therapeutic targets.

However, we also note that current research progress has significant limitations, which constrain the translation of tRNA modification-related therapeutic strategies from basic discoveries to clinical applications. On the one hand, most current investigations of tRNA modifications focus on single modification types, with little attention paid to multiple modifications on the same tRNA and to the crosstalk among various tRNA modifications. On the other hand, insufficient detection accuracy prevents precise quantification of tRNA modifications, and these two issues appear to be causally linked to some extent. Nevertheless, this has not hindered research progress. Recently, a class of non-coding small RNAs derived from tRNA has been discovered. These RNAs retain portions of the precursor tRNA’s sequence and structure, and are termed tsRNA modifications. These modifications are currently regarded as highly promising biomarkers and therapeutic targets for tumor screening, treatment, and prognosis. Whether the generation and abundance of tsRNAs correlate with immune system function remains to be explored.

Looking ahead, tRNA-modifying enzymes such as METTL1, as key molecules regulating the modification process, demonstrate potential as diagnostic biomarkers or therapeutic targets. However, large-scale clinical validation remains necessary before their clinical application [44]. This also indicates that therapeutic strategies targeting tRNA-modifying enzymes still have a long way to go before truly entering clinical practice. Furthermore, it is noteworthy that certain tRNA modifications capable of influencing CD4^+^ T cell states hold potential to trigger autoimmune diseases, particularly T cell-mediated conditions such as SLE [82]. Consequently, they may represent critical immune checkpoints in human resistance to autoimmune disorders.

In conclusion, delving deeper into tRNA modification will not only advance our understanding of epitranscriptomics but also hold promise for developing novel diagnostic and therapeutic strategies for intractable diseases such as IBD, cancer, and autoimmune disorders.

## Figures and Tables

**Table 1:** Regulators in the three major immune functions and diseases they cause.

Immune Function	Modification	Regulators	Mechanism	Associated Diseases	Research Model	References
Immune defense	Queuosine	QTRT1	Inactivation of QTRT1 leads to a decrease in Q-tRNA levels, resulting in upregulation of claudin-2 and downregulation of β-catenin and claudin-5, which inhibits epithelial cell proliferation and impairs TJ integrity.	IBD	patient samples, animal models, human intestinal epithelial cell culture	[16]
	Gm18	TrmH	Gm18-modified tRNA reduces TLR7 binding affinity, consequently weakening the activation of NF-κB and MAPK signaling pathways, suppressing IFN-α production in pDCs, and facilitating bacterial immune evasion.	infectious disease	human pDCs culture, in vitro biochemical evidence	[23,27]
	I34	ADAT2, ADAT3	By expanding the codon recognition spectrum, this modification facilitates efficient translation of antibody genes, sustains high-output antibody synthesis in B cells, and thereby enhances the body’s immune defense capacity against pathogens.	common variable immunodeficiency (CVID)	animal models, in vitro B cell assays, hypothesized association with CVID	[30]
Immune surveillance	m^7^G	METTL1	Mediates tumor immune evasion via the mTOR signaling pathway, leading to immunosuppression.	HCC, ICC, PCa, ESCC, HNSCC	patient samples, animal models, cancer cell culture	[39,44,45,52]
	m^1^A, m^3^C	ALKBH3	ALKBH3-mediated tRNA demethylation increases susceptibility to ANG cleavage, generating tsRNAs that bind and inhibit cytochrome c release, thereby blocking apoptosis and promoting tumorigenesis.	no established disease association	in vitro enzymatic assays	[55]
	Ψ50	PUS7	PUS7-mediated tRNA Ψ50 modification suppresses the TYK2-STAT1 pathway, thereby impairing NK/T cell function and promoting tumor immune evasion in GBM.	Glioblastoma multiforme (GBM)	glioblastoma stem cell culture	[60]
Immune homeostasis	6TG	TGT, HPRT	Selectively restricts differentiated effector cells, spares quiescent and naïve proliferating cell populations, and inhibits the activity of autoreactive T cells.	MS	animal models, T cell culture	[69]
	m^1^A58	TRMT61A, TRMT6	TRMT61A deficiency reduces tRNA m^1^A58 modification, consequently impairing MYC translation, downregulating CDK2 and cyclin E expression, and ultimately inducing S-phase arrest and proliferation suppression in CD4^+^ T cells.	T cell-related autoimmune diseases such as SLE	CD4^+^ T cell culture, hypothesized association with SLE based on indirect evidence	[79,80]
	Ψ55	TRUB1	TRUB1 deficiency impairs tRNA Ψ55 modification, leading to mitochondrial dysfunction, DAMP release, and exacerbated inflammatory responses in rheumatoid arthritis.	RA	hypothesized association with RA	[85]
	mcm^5^S^2^	Elp3, Ctu1	Elp3/Ctu1-mediated U34 (mcm^5^s^2^U) tRNA modification enhances Ric8b translation and activates mTORC2, thereby suppressing M1 polarization while promoting M2 polarization in macrophages.	experimental colitis	animal models, macrophage polarization assays	[89]

Note. The content of this table is a collation and summary of the relevant research content of this article.

## Data Availability

No new data were created or analyzed in this study. Data sharing is not applicable to this article.

## Abbreviations

The following abbreviations are used in this manuscript.

TJs	tight junctions
IBD	inflammatory bowel disease
QTRT1	queuine tRNA-ribosyltransferase catalytic subunit 1
TLR	Toll-like receptor
2′-O-Me	2′-O-methylation modifications
*E. coli*	*Escherichia coli*
I34	tRNA anticodon wobble position
wobble position	position 34
I	inosine
METTL1	methyltransferase-like 1
WDR4	WD repeat domain 4
m^7^G	N7 methylation of guanosine
RPTOR	regulatory-associated protein of mammalian target of rapamycin
HCC	hepatocellular carcinoma
PD-1	programmed cell death protein-1
pULK1	ULK1 phosphorylated at Ser758
ULK1	Unc-51-like autophagy-activating kinase 1
PMN-MDSCs	polymorphonuclear myeloid-derived suppressor cells
TIME	tumor immune microenvironment
PCa	prostate cancer
5′TOGs	5′-terminal oligoguanine-containing tRNA fragments
TME	tumor microenvironment
TAMs	Tumor-associated macrophages
m^1^A	N1-methyladenosine
m^3^C	3-methylcytidine
Cyt c	cytochrome c
TYK2	Tyrosine kinase 2
JAK	Janus kinase
IFNs	interferons
PUS7	pseudouridine synthase 7
6TG	6-thioguanine
TGT	tRNA guanine transglycosylase
CDK2	cyclin-dependent kinase 2
